# The left venous anatomy of the female pelvis and clinical outcomes: new insights

**DOI:** 10.1590/1806-9282.0711EDIT

**Published:** 2025-03-17

**Affiliations:** 1 Laboratório de Ginecologia Estrutural e Molecular (LIM/58), Disciplina de Ginecologia, Departamento de Obstetrícia e Ginecologia, Hospital das Clínicas, Faculdade de Medicina, Universidade de São Paulo – São Paulo (SP), Brazil.

Female chronic pelvic pain is indeed a huge challenger for the gynecologist. This disease requires awareness of several possible etiologies, both gynecological and non-gynecological, and may require expertise in diagnosis that goes beyond organic pain of organs located in the pelvis and multidisciplinary team effort for the best treatment in the search for long-lasting results^1^.

The knowledge of vascular anatomy of the female pelvis is essential to clarify the etiology, diagnosis, and treatment of diseases related to impairment of the venous system and its correlation with conditions that affect the female pelvis. The principal vessels supplying the female pelvic genital organs are the uterine arteries from the internal iliac vessels and the ovarian arteries which stem directly from the aorta. Most blood drainage from the pelvis is done via the internal iliac vein, which is a tributary of the inferior vena cava. The blood supply to the ovaries comes directly from the ovarian artery, a direct branch of the aorta. The venous drainage is unique as the right ovarian vein drains directly into the inferior vena cava, whereas the left renal vein drains the left ovarian vein^2^. In this context, the mechanism of the disorders of the pelvic vessel drainage system is important for the gynecologist. Pelvic varicose veins can arise due to three main anatomical alterations, such as nonphysiological drainage of the left ovarian vein into the ipsilateral renal vein, or by two sites of extrinsic venous stenosis, such as compression of the left iliac vein by the right iliac artery (Cockett's syndrome) or compression of the left renal vein by the superior mesenteric artery at the mesenteric aortic angle (Nutcracker syndrome)^3,4^.

The presence of one or more of the factors listed above determines the appearance of venous congestion in the pelvic territory^4^. It is important to note that these anatomical alterations mentioned occur mainly on the left side of the female pelvis. In fact, the venous environment resulting from pelvic congestion is responsible for the concentration of local inflammatory factors that can trigger oxidative stress, which can contribute as another factor related to the origin of endometriosis^5^.

In this sense, women with chronic pelvic pain secondary to pelvic congestion syndrome (PCS) report symptoms similar to endometriosis, which is one of the main differential diagnoses. However, it should be noted that diseases overlap, and, thus, endometriosis and PCS may be associated with the same patient and responsible for the symptoms^5^.

Araujo et al.^6^, in 2021, described that the incidence of ovarian endometriomas in the left adnexal region is higher when compared to the right side, in agreement with authors who describe the environment resulting from PCS as conducive to the development of endometriomas due to high concentrations of local inflammatory factors^5^. It may explain the confusion between PCS and endometriosis.

Another interesting clinical situation relates deep vein thrombosis and pelvic congestion in women. Rudolf Virschow (1821–1902) described in his triad the main etiological factors that determine venous thrombosis: hypercoagulability, endothelial injury, and blood stasis. With regard to stasis, we point out that most deep vein thromboses occur in the left lower limb, warning gynecologists that understanding the compression mechanism of the left pelvic venous drainage is crucial for an accurate diagnosis and, more than that, guiding patients on how to prevent thrombotic events in this situation^7,8^.

The importance of PCS in the gynecology setting and the formation of a multidisciplinary team are essential for a better understanding of this disease. A well-prepared anamnesis is sufficient for the inclusion of PCS as a cause of chronic pelvic pain. Endometriosis represents the main differential diagnosis, and more studies should be carried out to evaluate the concomitance of diseases.

## References

[1] Gibran L, Gonçalves BMM, Baracat EC, Soares JM (2024). The challenges of female chronic pelvic pain. Rev Assoc Med Bras (1992).

[2] Basile A, Failla G, Gozzo C (2021). Pelvic congestion syndrome. Semin Ultrasound CT MR.

[3] Champaneria R, Shah L, Moss J, Gupta JK, Birch J, Middleton LJ (2016). The relationship between pelvic vein incompetence and chronic pelvic pain in women: systematic reviews of diagnosis and treatment effectiveness. Health Technol Assess.

[4] Nasser F, Cavalcante RN, Affonso BB, Messina ML, Carnevale FC, Gregorio MA (2014). Safety, efficacy, and prognostic factors in endovascular treatment of pelvic congestion syndrome. Int J Gynaecol Obstet.

[5] Pacheco KG, Fortes Oliveira MR (2016). The prevalence of ovarian varices in patients with endometriosis. Ann Vasc Surg.

[6] Araujo RSDC, Maia SB, Lúcio JD, Lima MD, Ribeiro HSAA, Ribeiro PAAG (2021). Mapping of endometriosis in patients with unilateral endometrioma. Medicine (Baltimore).

[7] Harbin MM, Lutsey PL (2020). May-Thurner syndrome: history of understanding and need for defining population prevalence. J Thromb Haemost.

[8] Silver GA (1987). Virchow, the heroic model in medicine: health policy by accolade. Am J Public Health.

